# Knowledge and attitudes of undergraduate dental students at the University of Barcelona regarding antiresorptive and antiangiogenic medications: A cross-sectional study

**DOI:** 10.4317/jced.62092

**Published:** 2024-10-01

**Authors:** Karla Fuentes-Cazar, Jorge Toledano-Serrabona, Fabio Alves, Alba Sánchez-Torres, Rui Figueiredo, Cosme Gay-Escoda, Eduard Valmaseda-Castellón

**Affiliations:** 1 Oral Surgery and Implantology. Faculty of Medicine and Health Sciences, Universitat de Barcelona, Barcelona, Spain; 2 IDIBELL (Bellvitge Biomedical Research Institute), Barcelona, Spain; 3 Stomatology Department - A.C. Camargo Cancer Center, Sao Paulo, Brazil; 4 Teknon Medical Center, Barcelona, Spain

## Abstract

**Background:**

This study was carried out to determine the knowledge and attitudes of dental students at the University of Barcelona (Spain) concerning antiresorptive and antiangiogenic medications and their implications in dental treatment.

**Material and Methods:**

A cross-sectional study was conducted among dentistry students at the University of Barcelona using a 27-item questionnaire. This anonymous survey gathered demographic variables and assessed student interest in attending an educational session on oral pathology. Descriptive and bivariate analyses were performed following data collection.

**Results:**

A total of 105 students were surveyed. They all demonstrated awareness of antiresorptive medications, their pharmacokinetics and indications. Less than half, however, could identify drugs linked to osteonecrosis of the jaws (ONJ) or associated risk factors for oral complications. The students had a better understanding of antiresorptive drugs in comparison with antiangiogenic medications. Moreover, comparative analysis revealed that 5th year dental students had a higher level of knowledge of the dental implications of these drugs.

**Conclusions:**

This study shows that dental student knowledge of antiresorptive and antiangiogenic medications needs to be improved. The development of educational strategies to address the implications of these drugs in dental treatment is clearly indicated.

** Key words:**Medication-Related Osteonecrosis, Antiresorptive Drugs, Antiangiogenic Therapy.

## Introduction

Bone remodeling is a continuous process that is essential for maintaining bone integrity (1). It involves the coordinated activity of osteoclasts and osteoblasts within the bone remodeling unit (BRU) (2,3). Disease conditions can significantly accelerate bone resorption. Several antiresorptive drugs such as bisphosphonates (BPs), selective RANKL inhibitors (denosumab) and sclerostin inhibitors (romosozumab) have been developed to mitigate this imbalance in bone metabolism and prevent complications (4-8).

Antiangiogenic drugs in turn inhibit new blood vessel formation, and are basically used in cancer treatments. Evidence linking antiangiogenic medications to osteonecrosis of the jaws (ONJ) is limited and predominantly comes from case series (9,10).

Medication-related ONJ (MRONJ) has become one of the most serious complications associated with medication of this kind (9-11). The American Association of Oral and Maxillofacial Surgeons (AAOMS) defines MRONJ as persistent exposed bone in the maxillofacial area without healing for at least 8 weeks, in patients treated with antiresorptive or antiangiogenic drugs, in the absence of irradiation to the area (12).

The risk of MRONJ is influenced by various factors, including treatment duration, dosage, administration frequency, drug potency, and concurrent medications apart from antiresorptive or antiangiogenic treatments (13). The primary parameter for assessing ONJ risk is the therapeutic indication of the medication, such as cancer or osteoporosis / osteopenia (11). The current literature confirms a higher risk of developing MRONJ in cancer patients on antiresorptive therapy than in individuals treated for osteoporosis. While spontaneous ONJ can occur, dental extractions, periodontal or peri-implant disease, or invasive procedures are the most common triggering factors (5,6,10,14).

Patients taking antiresorptive drugs typically lack awareness of the possible complications that may occur after certain dental treatments (15). Therefore, dentists must compile a thorough medical history, inquiring into all medical aspects of the patient. Several studies evaluating knowledge of BPs and ONJ among dental students and professionals have yielded concerning results (16-20). The findings suggest that a significant number of students and dentists are unfamiliar with BPs and their dental treatment implications. Consequently, the present study was carried out to assess the understanding and attitudes of dental students at the University of Barcelona (Spain) regarding the dental implications of antiresorptive and antiangiogenic medications.

## Material and Methods

A cross-sectional study was conducted involving a cohort of dental students at the University of Barcelona. The study was designed according to the Strengthening the Reporting of Observational Studies in Epidemiology (STROBE) statement guidelines (21). The study was approved by the Clinical Research Ethics Committee of the Dental Hospital (protocol number 22/2022).

-Participants

The study included 4th and 5th year dental students enrolled in the 2022-2023 academic year who consented to participate. There were no exclusion criteria.

Prior to participation, the researcher explained the study objectives and methodology, and answered all the questions of the participants. Students were advised of their right to decline to participate in the study. Written informed consent was obtained before initiating the survey.

-Settings

A 27-question survey was developed to evaluate knowledge of antiresorptive and antiangiogenic medications. The first part of the questionnaire gathered demographic data, while the subsequent 16 questions assessed understanding of the medications, encompassing aspects such as local risk factors, medical indications, mechanisms of action, active ingredients, commercial names, preventive measures, and the treatment of complications. The last set of questions measured interest in receiving additional education in the field of oral pathology. The questionnaires were provided by a single researcher and were completed without access to external information sources, maintaining participant anonymity.

-Variables and measurements

The participants completed a validated questionnaire, which was a modified version of the instrument used by De Lima *et al*. (17). Additional questions were included to address other medications known to cause ONJ, such as selective RANKL inhibitors and antiangiogenic drugs. A comprehensive descriptive analysis was undertaken following data collection.

The assessment yielded scores out of a potential 16 questions, based on responses structured as dichotomous variables.

-Sample size and statistical analysis 

Data were recorded using a Microsoft Excel® spreadsheet (Microsoft Corp., Redmond, Washington, USA). For the statistical analysis concerning the knowledge of antiresorptive and antiangiogenic medications, each question was assigned a value of one point. In questions with a single response, a point was awarded only in the case of a correct answer. When multiple-choice questions were involved, correct responses were deemed accurate only when all options were selected correctly.

Statistical analysis was performed using Stata15.1 (StataCorp®, College Station, TX, USA). The normal distribution of quantitative variables was assessed using the Shapiro-Wilk test, complemented with box plot analyses. Descriptive and bivariate analyses using the Student t-test were applied to examine differences in means of correct answers among the different courses of the Dentistry degree. Statistical significance was set at *p* < 0.05.

## Results

A total of 105 responses were obtained from a total of 180 4th and 5th year dental students (58.3%): 82 women (78.1%) and 23 men (21.9%), with an average age of 24.4 years (standard deviation [SD] ± 3.4). Fifty-seven students (54.3%) were from the 4th year and 48 (45.7%) were from the 5th year. Both groups indicated that their primary source of information about the medication was through the university (88.6%), followed by the internet. Forty-two participants (40%) reported having prior education on the topic.

 Table 1 shows the distribution of responses corresponding to each question in distinct categories, delineating the count and percentage of correct, partially correct, and “Don’t know” responses. The findings imply diverse levels of knowledge across various topics within the surveyed categories.

Eighty-three participants (79%) claimed to be familiar with antiresorptive medication. However, less than half were able to recognize the drugs that can cause MRONJ or the associated risk factors that could lead to side effects in the oral cavity.

**Figure 1:**
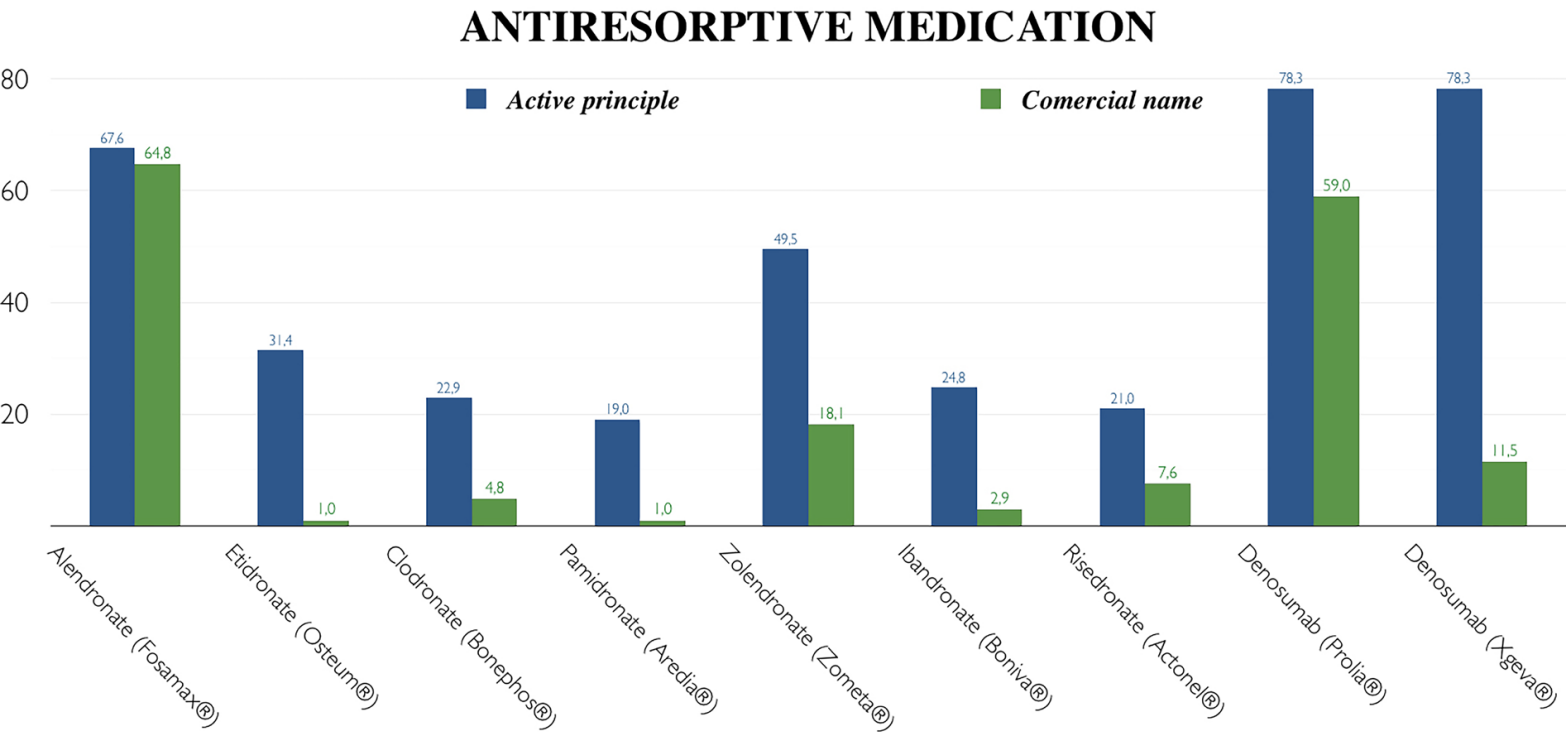
Comparative knowledge analysis: active ingredients versus commercial names of antiresorptive medication.

Given the crucial importance of correctly identifying medications, a comparison was made between knowledge of the active ingredients and the commercial brand names. Participants seemed to recognize the most commonly used medications on the market, such as alendronate (70.8%), zolendronate (52.1%) or denosumab (78.3%). Nevertheless, there was generally more confusion in recognizing commercial names, as observed with Fosamax® (64.5%), Zometa® (18.8%) or Prolia® (58.3%) / Xgeva® (11.5%) (Fig. 1).

**Figure 2:**
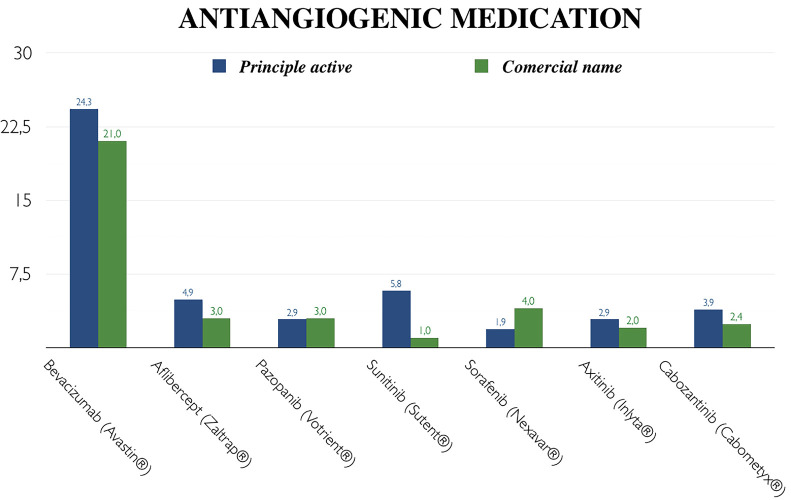
Comparative knowledge analysis: active ingredients versus commercial names of antiangiogenic medication.

Knowledge regarding antiangiogenic medication was poorer. Seventy percent of the participants recognized not knowing such drugs. As shown in Figure 2, the most frequently identified active ingredient - with only 25 responses (23.4%) - was bevacizumab. The commercial name (Avastin®) was recognized by only 21 (21%) students.

**Figure 3:**
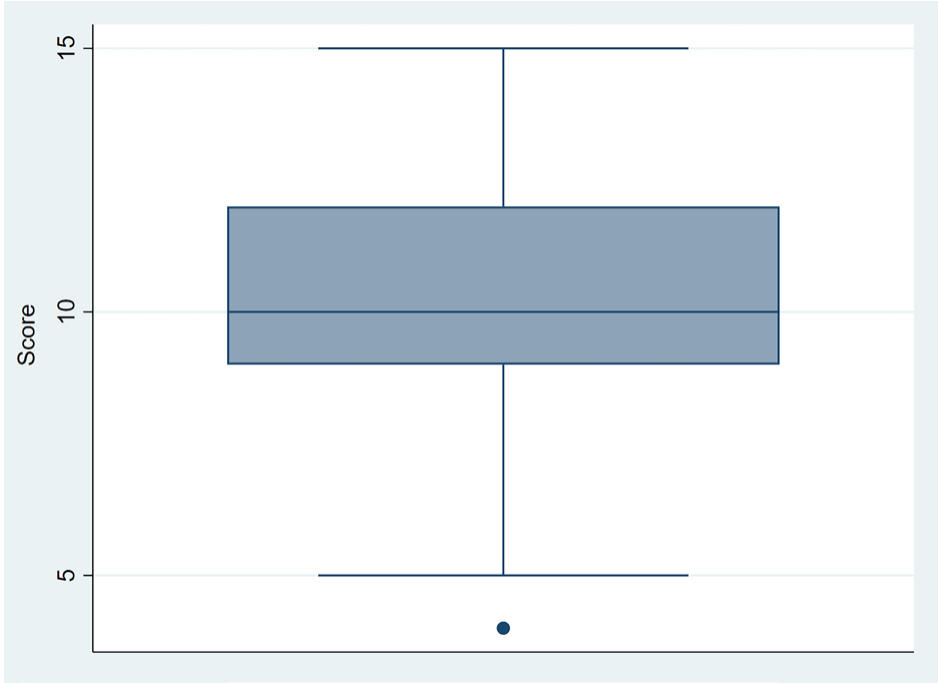
Box plot analysis: distribution of scores in the total student sample.

**Figure 4:**
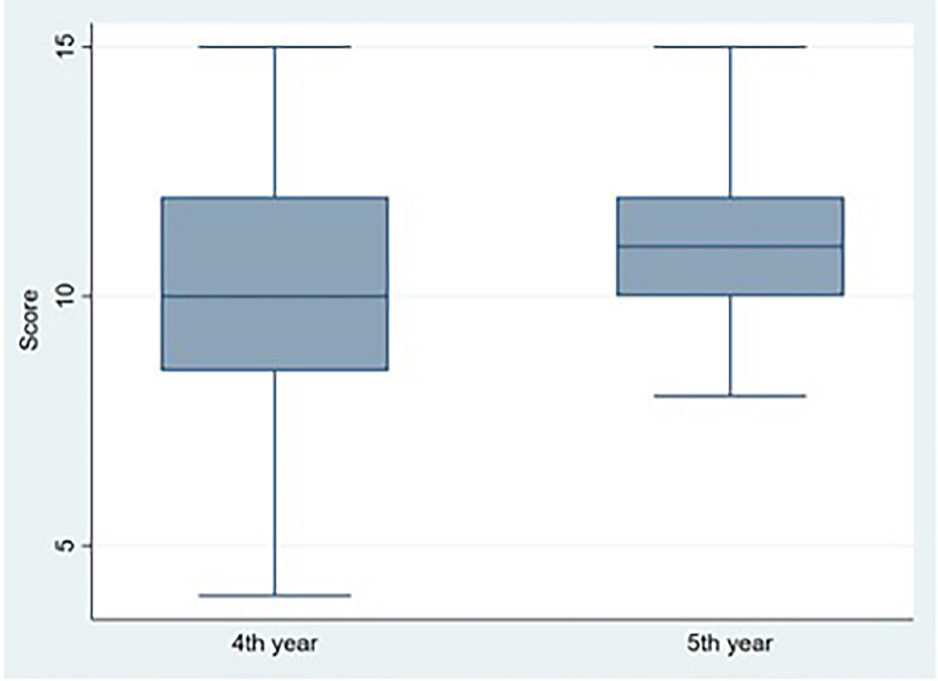
Knowledge distribution assessment: box plot analysis according to academic year.

Figure 3 shows the box plot of the total scores obtained in the survey. Fifty percent of the scores fell within the range of 9 to 12, out of a possible 16. The box plot shown in Figure 4 suggests that 5th year dental students displayed higher results, though without statistically significant differences (*p*=0.07).

All students were willing to attend oral medicine courses or congresses. Twenty-eight participants (26.7%) had never attended an oral pathology lecture, but the great majority of individuals (95 students; 90.5%) expressed interest in participating in future oral pathology workshops.

## Discussion

The objective of this study was to assess the knowledge and attitudes of dental students at the University of Barcelona regarding antiresorptive and antiangiogenic medications and their impact on dental treatments. The results indicate that students have a lack of understanding concerning these medications and their implications in dental care.

One of the limitations of the present study is related to the absence of a group of active dental practitioners, which limits generalization of the results to a real-life clinical setting. Thus, comparative studies involving dental professionals should be made to provide a more comprehensive view of the knowledge of dentists in relation to this particular topic.

The findings of this study are in line with those of other studies that have assessed knowledge about BPs and ONJ among dental students and dentists, revealing concerning results. In 2009, Lopez-Jornet *et al*. (16) conducted a study on the awareness of antiresorptive medication among students and dentists in the region of Murcia (Spain). Their results highlighted that dental students and practicing dentists had doubts regarding the management of invasive dental procedures (such as tooth extraction) in patients taking BPs. Only 8 students and 20 dentists were aware of the appropriate procedure once the diagnosis was established.

Likewise, in 2014, De Lima *et al*. (17) conducted a similar study in a Brazilian population and noted a rise in cases of ONJ associated with BP use. While 43.3% claimed to have knowledge, most could not identify any active ingredient or commercial name. Only 15% recognized alendronate and its commercial name.

In a broader context, Alhussain *et al*. (19) carried out a cross-sectional study in North America designed to examine the knowledge, practices, and opinions of 1579 dentists. The survey employed the 2009 position paper from the AAOMS, which provided insights into the risks associated with the development of BP-related ONJ (BRONJ). Within this extensive dentist population, only 23.8% demonstrated proficiency in adhering to these guidelines, and 49.7% were not comfortable treating patients with BRONJ.

Our results are also in line with recent European-based publications, including studies conducted in Italy, Croatia and Germany (22-24), where limitations were found in both dental students and practicing dentists when assessing knowledge related with MRONJ.

On the other hand, the present study adds new data to the literature, since it includes antiangiogenic drugs. The observed lack of knowledge about this group of drugs is considerably greater and very concerning, suggesting a need to increase educational resources on this topic. Also, an effort was made to evaluate knowledge related to antiresorptive therapies (such as BPs and RANKL inhibitors). The existing literature highlights an increased risk of complications when patients use multiple medications for different indications. Thus, it is essential to provide this information to clinicians. Zhao *et al*. (25) showed a synergic relationship between antiangiogenic drugs and antiresorptive drugs in MRONJ. Indeed, these authors indicated that while antiangiogenic drugs alone may not cause severe MRONJ, they can intensify its impact by amplifying the inhibitory function of gingival fibroblasts influenced by antiresorptive drugs.

Tooth extractions represent one of the most common dental procedures and are a primary risk factor for the development of MRONJ (6). Consequently, it is crucial to provide information to both patients and dental professionals with the aim of increasing awareness and assurance in the effective management of MRONJ complications. This, in turn, contributes to a more confident and composed approach by dental professionals in treating such complications.

The present outcomes seem to underline the need to integrate targeted modules, workshops, and case studies on antiresorptive and antiangiogenic medications into dental curricula. Awareness campaigns and the dissemination of guidelines in dental schools and professional organizations also seems necessary. On the other hand, interdisciplinary collaboration between dental and other healthcare professionals that might prescribe these medications could be important for improving patient care.

While our primary findings were based on a small population of dental students, consistent outcomes indicating a lack of knowledge about these medications have been observed in similar studies conducted in different communities. Therefore, addressing this educational gap among our students within the academic sphere is paramount. These proposed interventions and considerations might help improve the overall preparedness of dental professionals in managing patients under antiresorptive and antiangiogenic treatments.

In conclusion, based on the results obtained and considering that the sample corresponds to a single Spanish university, dental students appear to recognize the medication but show insufficient knowledge about crucial aspects for preventing MRONJ.

The data indicate that dental students at the University of Barcelona may be faced with challenges in recognizing some drugs associated with this serious complication when compiling the medical history. It is important to stress that all participants were interested in undergoing additional training on these topics.

## Figures and Tables

**Table 1:** Knowledge assessment of the survey and distribution of responses. n = number of responses.

Category	Question	Answers n (%)		
		Correct	Partially correct	Don't know
Anamnesis	During the anamnesis, do you believe it is important to consider whether the patient has been or is currently undergoing treatment with bisphosphonates?	105 (100)		
	During the anamnesis, do you believe it is important to consider whether the patient has been or is currently undergoing treatment with RANKL inhibitors (denosumab) medication?	103 (98.1)		2 (1.9)
	During the anamnesis, do you believe it is important to consider whether the patient has been or is currently undergoing treatment with antiangiogenic medication?	102 (97.1)		3 (2.9)
Antirresorptive medication	What are the medical indications for the use of bisphosphonates?	81 (77.1)	20 (19.1)	4 (3.8)
	What is the principal mechanism of action for the medication?	89 (84.8)		16 (7.8)
	Please identify the bisphosphonates that you are familiar with:	41 (39.1)	60 (57.1)	4 (3.8)
	What commercial names represent medications from the antiresorptive drug family?	75 (71.4)	13 (12.4)	17 (16.2)
	Are you aware of any side effects appearing in the oral cavity caused by antiresorptive medication?	97 (92.4)		8 (7.6)
	If known, please check which side effect(s) is(are) caused by this group of medications:	44 (41.9)	55 (52.4)	6 (5.7)
Antiangiogenic medication	What are the medical indications for the use of antiangiogenic medication?	47 (44.8)	36 (34.3)	22 (21)
	What is the principal mechanism of action for the medication?	82 (78.1)		23 (21.9)
	Please identify the antiangiogenic medication that you are familiar with:	15 (14.3)	54 (51.4)	36 (34.3)
	What commercial names represent medications from the antiangiogenic drug family?	32 (30.5)		73 (69.5)
Risk factors and treatments	What risk factors associated with these medications can lead to side effects in the oral cavity?	36 (34.3)	66 (62.9)	3 (2.9)
	What dental procedures and oral diseases can be risk factors for the development of side effects in the oral cavity in patients taking antiresorptive or antiangiogenic medication?	75 (71.4)	26 (24.8)	4 (3.8)
Prevention	What care and/or procedures can be implemented to prevent the occurrence of side effects?	82 (78.1)	17 (16.2)	6 (5.7)

## Data Availability

The datasets used and/or analyzed during the current study are available from the corresponding author.
